# PROFILE OF COMMUNITY-DWELLING OLDER ADULTS ONE YEAR INTO COVID-19

**DOI:** 10.1093/geroni/igac059.1803

**Published:** 2022-12-20

**Authors:** Helen Lach, Devita Stallings, Janice Palmer, Rebecca Lorenz

**Affiliations:** Saint Louis University, St. Louis, Missouri, United States; Saint Louis University, St. Louis, Missouri, United States; Webster University, St. Louis, Missouri, United States; University at Buffalo, Buffalo, New York, United States

## Abstract

We surveyed community-dwelling older adults in 2020, primarily in the Midwest, to learn about their experiences during the initial months of the COVID-19 pandemic. In summer 2021, we collected quantitative and qualitative one-year follow-up data on 118 participants from the original cohort who agreed to be contacted later (n=246; 48% response rate). Respondents included mostly women (75.2%), White (95%) with a mean age of 76.4 years and 15 mean years of education; half were married (49.5), and 40.7% lived alone. Compared to 2020, participants had a higher mean score on the CESD10 (9.76 vs. 6.97); although self-rated health and quality of life scores were similar (94-95% good-excellent). Mean scores on the Brief Resilient Scale were similar. In this group, 99.3% had been vaccinated, and had a concern score of 3.7 out of 5 that there would be more COVID outbreaks. Themes from their reported adaptations during the pandemic included taking steps for physical and mental self-care, maintaining connections with others, and finding credible sources of information. Connecting with others online was the biggest technological change during this time, and the computer was the most valued technology. These educated, middle class older adults continued to adapt to the COVID-19 pandemic, but those who did not respond to the second survey may have had more health or mental health challenges. These findings suggest a need for health care providers to screen older adults for depression so that early interventions can take place.

